# Penile Fracture with Associated Urethral Rupture

**DOI:** 10.1155/2010/791948

**Published:** 2010-11-07

**Authors:** Nicholas A. Boncher, Gino J. Vricella, Jason T. Jankowski, Lee E. Ponsky, Edward E. Cherullo

**Affiliations:** ^1^Department of Urology, University Hospitals - CASE Medical Center, Cleveland, OH 44106-4931, USA; ^2^Department of Urology, University Hospitals of Cleveland, Case Western University School of Medicine, Cleveland, OH 44106-4970, USA

## Abstract

Penile fracture of the erect penis is an uncommon but emergent urological trauma. Potential outcomes include erectile dysfunction, penile curvature, and urethral injury. Treatment is emergent surgical repair. We present the case of a 42-year-old man with a penile fracture complicated by a urethral rupture and subsequent repair. A discussion of the key aspects of this condition is presented.

## 1. Introduction

Penile “fracture”—rupture of the corpus cavernosum secondary to trauma to the erect penis—is an uncommon but emergent urologic condition. It is as infrequent as 1 in 175,000 hospital admissions in the United States [1] although reports indicate that it is more common abroad [2]. In the US, most cases occur when the erect penis is struck against the symphysis or perineum after the penis has slipped out of the vagina during aggressive intercourse [3]. Herein, we report a rare case of a penile fracture with urethral rupture.

## 2. Case Report

**Figure 1:**
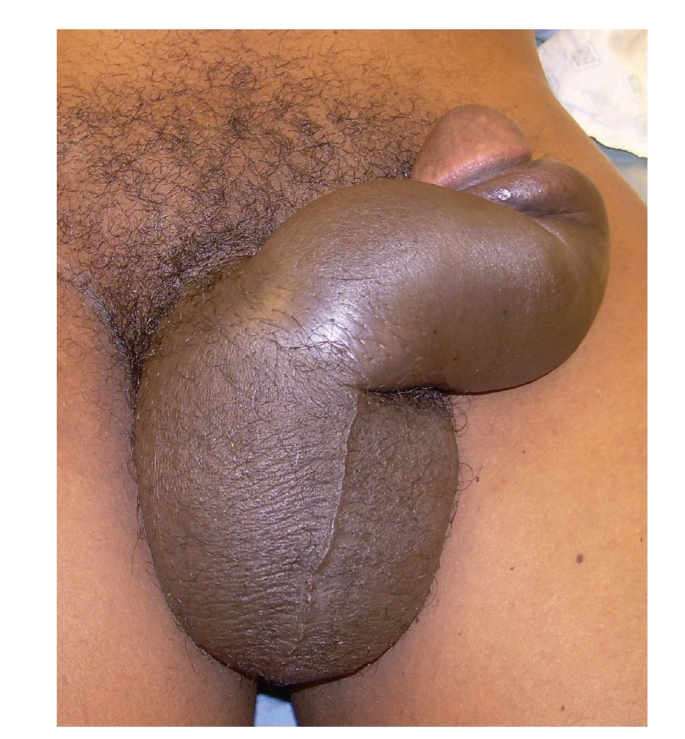
Preoperative examination.

A 42-year-old man reported to the emergency room approximately 8 hours after striking his erect penis against his partner's perineum during sexual intercourse. This was accompanied by immediate pain and detumescence. Significant penoscrotal swelling ensued and the patient was unable to void beginning at the time of the injury. Physical exam revealed a swollen, ecchymotic penis without blood at the meatus (see Figure 1). Leftward penile deviation was noted opposing the right-sided eggplant hematoma on the penile shaft. Appropriate concern for penile fracture led to operative management. 

**Figure 2:**
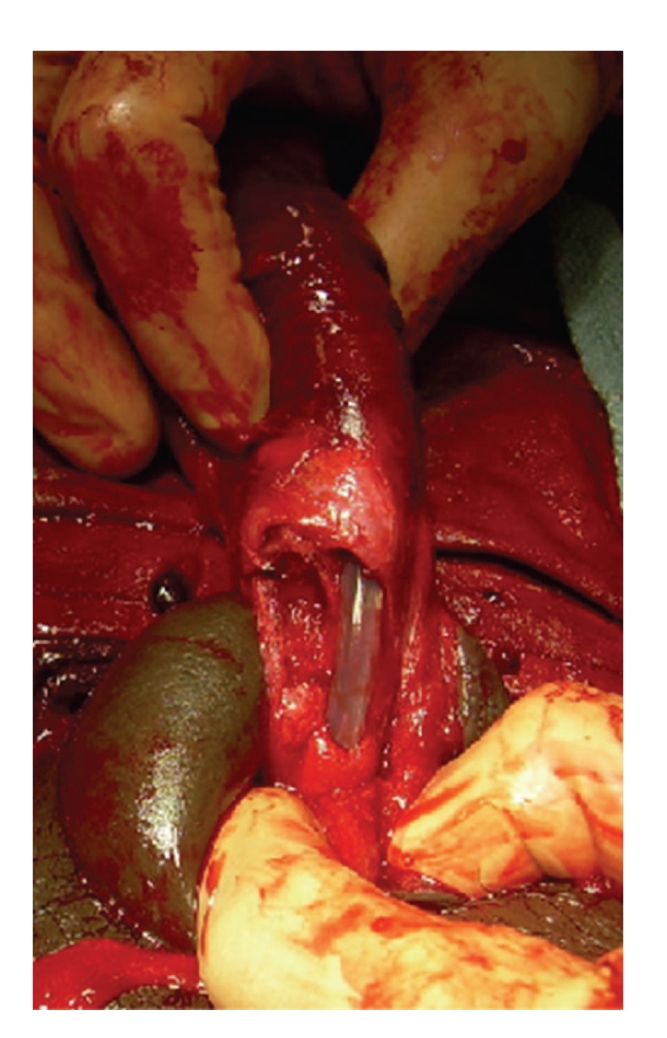
Intraoperative findings: right cavernosal tear and urethral rupture (foley catheter in urethra).

**Figure 3:**
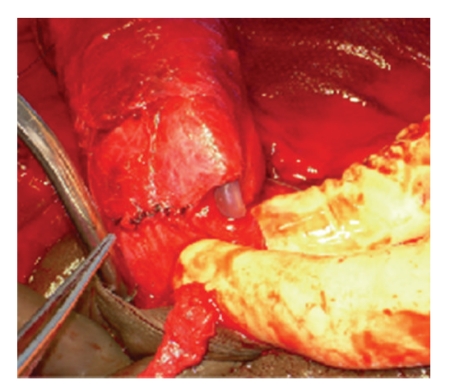
Cavernosal repair.

**Figure 4:**
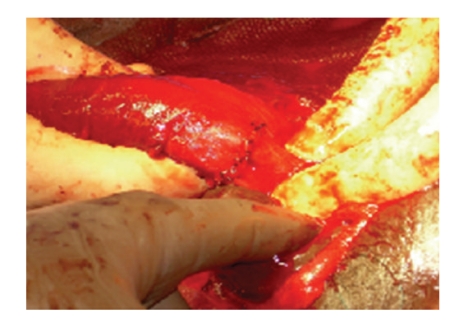
Completed repair.

**Figure 5:**
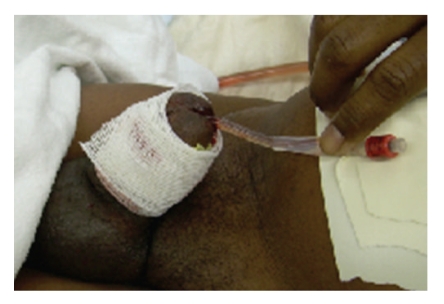
Post-operative appearance.

At the time of operation, a subcoronal circumcising incision was made and the penis was degloved. After evacuation of a large hematoma, a significant defect was identified in the right corpus cavernosum. The defect was ventral in location though lateral of midline. Additionally, a large injury involving the ventral half of the urethra was identified (see Figure 2). An 18 French catheter was introduced through the meatus, maneuvered across the defect and into the bladder. The urethra was then spatulated and closed in a watertight fashion over the catheter using 5-0 PDS suture. The cavernosal injury was repaired in an interrupted fashion using 5-0 PDS suture (see Figure 3). We have found this to be of adequate strength if done in an interrupted fashion with a several week abstinence of intercourse although 3-0 PDS is likely a more preferable suture choice for most situations. A circumcision was then performed and the skin edges were reapproximated using interrupted 4-0 Vicryl suture (Figures 4 and 5). Postoperatively, the patient received diazepam 5 mg nightly in an attempt to prevent nocturnal erections. The urethral catheter was left in place for 4 weeks and then removed. The patient voided without difficulty and was able to regain erectile function. There has been no development of penile curvature or erectile dysfunction since last followup visit four years following surgery.

## 3. Discussion

The increased risk of penile rupture during tumescence is partially due to the fact that the tunica albuginea stretches and thins when the penis is erect. Studies show that in the flaccid state, it is up to 2.4 mm thick, while in the erect state, it can be as thin as 0.25–0.5 mm [4]. If the tunica albuginea buckles, the resultant increase in intracavernosal pressure can lead to a tunical tear. This typically occurs during an attempted reentry in a position in which the weight and thrust of the partner is brought to bear directly onto the tip of the penis. Given the microanatomy of the penile shaft, the injury typically occurs along the ventrum of the corporal bodies. This area corresponds to a thinning of Buck's fascial layer as it splits with one lamella continuing to surround the corpora cavernosum and the second to invest the corpus spongiosum. In response to the increased pressure during the injurious insult, the pattern of injury reflects a blowout type of tear in the ventral corporal bodies. Associated urethral injuries are reported in anywhere from 1% to 38% of patients [3, 5]. Such a high degree of variability likely has a geographic component. In the Middle East, most penile fractures are caused by the patient forcefully bending the penis forward in an attempt to achieve detumescence—a practice referred to as “taghaandan” [5]. Thus, these injuries are “low energy,” as compared to penile fractures sustained during sexual intercourse. During the latter mechanism, the penis is typically bearing the entire load of the thrust and or partner's body weight during an errant reentry attempt. Thus, taghaandan injuries probably have less risk of associated urethral rupture given the lack of additional opposed force provided by the partner's perineum. Retrograde urethrography or cystourethroscopy should be considered during initial workup. However, this can be omitted if proceeding directly to operative repair, but in those cases, exploration for a urethral injury must then be performed intraoperatively. Signs of urethral injury include blood at the meatus, an inability to void, or hematuria. One should be aware that urethral bleeding without a urethral injury has been reported after penile fracture [6]. Additionally, false negative results have been reported with retrograde urethrography [7], so we continue with direct inspection of the urethra during surgical exploration regardless of negative retrograde urethrography. 

Reports have shown that urethral injuries can be closed in a spatulated, watertight fashion with subsequent urethral catheter drainage for at least three weeks [8]. Minimal urethral injuries or contusions may be managed with urinary diversion alone [9]. Postoperatively, patients should be counseled that there risk of erectile dysfunction remains high. This is related, at least in part to vascular anomalies of both the arterial inflow and venous outflow systems. Nearly 50% of patients will have anomalies on penile duplex ultrasound more than one year following surgical repair for penile fracture, with the majority of such abnormalities being cavernosal insufficiency or arterial insufficiency [10]. 

Regarding the corporal injury, studies have demonstrated that immediate surgical repair leads to faster recovery, less morbidity, and less penile deformity when compared to nonoperative management [11]. Penile curvature is present in less than 5% of cases and occurs most often in patients with a delayed presentation [6]. 

Regarding the use of diazepam as an aide to prevent erections during healing, this strategy is based on the work of Jallu and associates who used diazepam in conjunction with oxyphenbutazone to prevent erections in patients treated conservatively for penile fracture [12]. There is little prospective data regarding the use of this medication, and its incorporation in management schema should not necessarily be construed as standard of care in the modern day. Patients should be counseled to abstain from sexual activity for a period of at least 6–8 weeks [13].
